# Enhanced Stability and Mechanical Properties of a Graphene–Protein Nanocomposite Film by a Facile Non-Covalent Self-Assembly Approach

**DOI:** 10.3390/nano12071181

**Published:** 2022-04-01

**Authors:** Chunbao Du, Ting Du, Joey Tianyi Zhou, Yanan Zhu, Xingang Jia, Yuan Cheng

**Affiliations:** 1 College of Chemistry and Chemical Engineering, Xi’an Shiyou University, Xi’an 710065, China; duchunbao218@126.com (C.D.); duting102@126.com (T.D.); zhuyanan@xsyu.edu.cn (Y.Z.); jiaxingang76@xsyu.edu.cn (X.J.); 2 Institute of High Performance Computing, A*STAR, Singapore 138632, Singapore; joey_zhou@ihpc.a-star.edu.sg; 3 Monash Suzhou Research Institute, Monash University, Suzhou Industrial Park, Suzhou 215000, China; 4 Department of Materials Science and Engineering, Monash University, Clayton, VIC 3800, Australia

**Keywords:** graphene, nanocomposite film, film-forming ability, stability, mechanical properties

## Abstract

Graphene-based nanocomposite films (NCFs) are in high demand due to their superior photoelectric and thermal properties, but their stability and mechanical properties form a bottleneck. Herein, a facile approach was used to prepare nacre-mimetic NCFs through the non-covalent self-assembly of graphene oxide (GO) and biocompatible proteins. Various characterization techniques were employed to characterize the as-prepared NCFs and to track the interactions between GO and proteins. The conformational changes of various proteins induced by GO determined the film-forming ability of NCFs, and the binding of bull serum albumin (BSA)/hemoglobin (HB) on GO’s surface was beneficial for improving the stability of as-prepared NCFs. Compared with the GO film without any additive, the indentation hardness and equivalent elastic modulus could be improved by 50.0% and 68.6% for GO–BSA NCF; and 100% and 87.5% for GO–HB NCF. Our strategy should be facile and effective for fabricating well-designed bio-nanocomposites for universal functional applications.

## 1. Introduction

Two-dimensional (2D) nanomaterials have recently opened a new era for flexible devices owing to their exotic electronic and optical properties [1,2,3]. Graphene is an emerging constituent for 2D nanomaterials, and graphene films hold great potential for meeting various intellectualized functionalities [4,5,6]. However, the mechanical properties of pure graphene films have significant flaws, such as limited flexibility and stability [7,8]. Reinforcing components are usually added to produce nanocomposite films (NCFs) to improve overall characteristics, which opens new avenues for graphene’s use. Synthetic polymers are used in most graphene-based NCFs owing to their superior designability and usefulness [9,10]. However, synthetic polymers do not easily decompose naturally, resulting in considerable solid waste [11,12]. Therefore, the present trend is to develop environmentally friendly graphene-based NCFs to reduce carbon emissions and allow more recycling of materials.

Biomacromolecules (BMMs), which are indispensable for in vivo life, including proteins, polypeptides, enzymes, DNA, RNA, lipids, and polysaccharides, are being used in in vitro applications because of their exceptional functionality and biodegradability [13,14,15,16,17]. When included in NCFs with nanomaterials that have the desired photoelectric and thermal properties, multifarious applications are available, such as biosensors, artificial tissue, information storage, and drug delivery [18,19,20]. Various studies report that integrating BMMs and graphene has already been done, forming some novel composites will multiple applications [20,21,22,23,24,25]. For example, Liu et al. employed a simple method to prepare low-cost graphene and silk-based pressure sensor, which could be used as artificial skin to monitor the pressure of the human body in real-time [26]. In another case, Chu et al. fabricated a hybrid scaffold using graphene oxide (GO) and an acellular dermal matrix, promoting cell proliferation in the wounds of diabetics [27]. Recently, Chang et al. loaded a heat shock protein 90 inhibitor NVP-AUY922 on a GO-based GO/BaHoF5/PEG nanocomposite to perform sensitized photothermal therapy (PTT). The achieved nano-platform, GO/BaHoF5/PEG/NVP-AUY922, had excellent biocompatibility and made tumor cells more sensitive to hyperthermia, which could promote the development of low-laser-hazard PTT [28]. In the work of Zhao et al., photomodule single-layer reduced graphene oxide (rGO) has been organized into a well-defined multilayer stack with the help of amyloid-like protein aggregates [29]. The as-fabricated hybrid film reliably adheres to the plastic substrate with robust interfacial adhesion. The sensitive photothermal effect of rGO in the bilayer film can be initiated with a blue laser from 100 m away, indicating that the combination of GO with BMMs exhibited great potential in remote light control of robots and devices. Our previous work has summarized the bio–nanomaterial interaction mechanisms at the molecular level of some typical 2D nanomaterials and BMMs, including non-covalent and covalent interactions, and proposed the challenges for the future development of 2D materials and biomacromolecules [30]. Despite significant advances, insufficient attention has been devoted to the stability of graphene-based NCFs in applicable environments involving acidity, alkalinity, salt, heat, and so on. Furthermore, the production processes of NCFs with graphene and biomacromolecules at the molecular level, including the species, conformations, film-forming ability, and mechanical characteristics, need to be investigated further. As a result, there are still major opportunities in, and obstacles to, extending more general biomacromolecules, including understanding and controlling molecular pathways.

**Figure 1:**
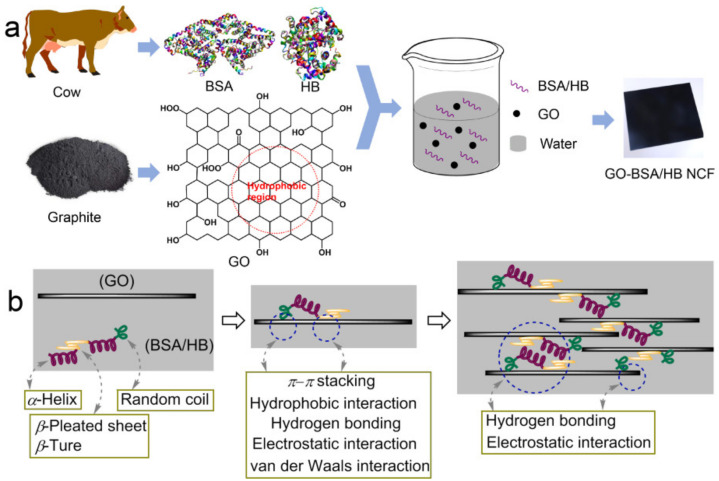
Scheme of self-assembly of GO with BSA/HB for GO–BSA and GO–HB NCF (**a**). Possible binding mechanism of a GO sheet with BSA/HB and the fabrication of a NCF (**b**).

In this work, after considering the desirability of simplicity, low cost, and reproducibility for BMMs to be used in scaled-up applications, ordinary and commercialized proteins, i.e., bovine serum albumin (BSA) and hemoglobin from bovine-blood (HB), were chosen for assembly with GO to fabricate NCFs (i.e., GO–BSA NCF and GO–HB NCF; Figure 1a). Using lysozyme (Lyz)-formed NCF (i.e., GO–Lyz NCF) for a comparison, the film-forming abilities of the NCFs were investigated comprehensively by tracking the experimental progress and analyzing the microstructures. X-ray photoelectron spectrometry (XPS), scanning electron microscopy (SEM), and circular dichroism (CD) spectroscopy, along with theoretical simulations, were adopted to reveal the binding mechanism. Moreover, the stability, thermostability, and mechanical properties were investigated by dissolution experiments, differential scanning calorimetry (DSC), and nanometer indentation. Although the structures and properties of BSA, HB, and Lyz are different, consistent stability and improved mechanical strength were achieved, which might provide some inspiration for fabricating other stable nanocomposites.

## 2. Materials and Methods

### 2.1. Experimental Materials

GO with a thickness of 1.2–5 nm and a lateral size of 50 nm to 3 μm was purchased from Hangzhou Nano-Mall Technology Co., Ltd., Hangzhou, China. BSA, HB, and Lyz were supplied by Macklin. NaCl (AR, >99.5%), HCl (AR, 36.0–38.0%), and NaOH (AR, >99.6%) were products of Sinopharm Chemical Reagent Co., Ltd., Shanghai, China.

### 2.2. Fabrication of NCF

The fabrication of NCF was conducted by using simple vacuum filtration. Typically, protein (i.e., BSA, HB, or Lyz) was dissolved in the deionized water to form the homogeneous solution with 4 mg mL^−1^. After that, the aqueous GO solution (4 mg mL^−1^, 5 mL) was added to the protein solution (5 mL) after the ultrasonic treatment of the aqueous GO solution at 100 W, at 20 °C, for 30 min. Afterward, the mixed solution was stirred at room temperature (26.4 °C) for 16 h to complete the self-assembly process. Then, the mixed solution was poured into the filter flask to remove the water and obtain the wettish NCF. After drying at 30 °C in a vacuum drying oven for 2 h, the dried GO–BSA, GO–HB, and GO–Lyz NCF were kept in a desiccator. The preparation method of the GO film was the same as that of the above NCFs without adding protein. Notably, the structure and characteristics of the composite films varied depending on the proportions of GO and protein [31]. During the experiments, other ratios of GO and protein were also tested, but the films obtained were all poor. When the amount of GO was higher than protein, the film was brittle. In the opposite case, the film was thin and difficult to remove from the substrate.

### 2.3. Analysis and Characterization

SEM (FEI Talos F200X) was used to observe the morphology and structure of each film at a high voltage of 10.0 kV. Chemical surface characterization was performed by XPS (Shimadzu Kratos, Manchester, United Kingdom) with monochromatic Al Ka radiation (1486.6 eV); the deconvolution method using Gaussian and Lorentz curve fittings was employed to conduct the semiquantitative analysis of the elements. The thermal properties of films were characterized by DSC (Nestal DSC214, Selb, Germany). The CD spectroscopy experiments were carried out using a CD spectrometer (Applied Photophysics Ltd. Chirascan, Leatherhead, UK); the GO–BMM compound solution after self-assembly was diluted by a factor of 27; CD spectra were obtained by scanning the diluted GO–BMM compound solution and deducting the background of the GO solution. Secondary structures of BSA and HB were determined by fitting the far-UV CD data using CDNN algorithms. An optical microscope (Olympus BX51, Tokyo, Japan) was used to observe the morphology of GO and GO–BMM solutions at room temperature. The mechanical properties of NCFs were characterized by a nanometer indentation instrument (UNHT) at room temperature (28.3 °C) with five random positions (RP) and the compression rate of 1 mm min^−1^; *F*_m_ (mN) and *h* (nm) were confirmed in the obtained curved; *H*_IT_ (GPa) and E* (GPa) were calculated by the supporting analysis software. The stability of films was measured in separate NaCl, HCl, and NaOH aqueous solutions, each with a concentration of 0.1 M.

## 3. Results and Discussion

### 3.1. Structural Property of NCFs

The fabrication of NCFs was conducted using simple vacuum filtration (Figure 1a). Protein (i.e., BSA, HB, and Lyz; see Figure S1a–c) was dissolved in the deionized water to form the homogeneous solution and then mixed with the ultrasonically treated GO aqueous solution. After stirring, the mixed solution was poured into a filter flask to remove the water and obtain the wettish film. Finally, the GO–BSA, GO–HB, and GO–Lyz NCFs were obtained after drying. The most spread out BSA, HB, and Lyz were in the three dimensions of the crystalline state was about 14 × 8 × 5 nm^3^, which was far less than the size of GO sheet (50 nm–3 μm) [32]. BSA, HB, and Lyz were more likely to be bound on the surface of a GO sheet rather than the edge. The interactions between the GO sheet and BSA/HB/Lyz were dominated by the multiple non-covalent interactions (Figure 1b). Notably, because protein molecules could not be trapped by the filter membrane, the pure protein film could only be obtained by solvent evaporation and could not be obtained using vacuum filtration. GO is a 2D material with a high aspect ratio that is useful for adsorbing protein molecules as a skeleton when creating films, and its outstanding mechanical strength is very advantageous [33,34]. To unravel the protein–GO interaction mechanism, specific experiments were designed, and the results obtained are discussed step by step as follows.

**Figure 2:**
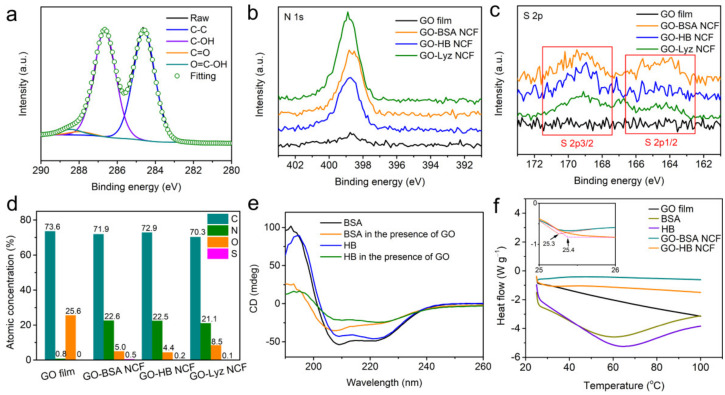
C 1s high magnification of films (**a**). N 1s high magnification of GO film (**b**). S 2p high magnification of films (**c**). The atomic compositions of films (**d**). CD spectra of BSA and HB with and without GO induction (**e**). DSC curves of GO film, BSA, HB, GO–BSA NCF, and GO–HB NCF with a heating rate of 5 °C min^−1^ in N_2_ flow from 25 to 100 °C (**f**).

The sizes (layers, transverse, and longitudinal direction) and properties (functional groups and groups density) of GO were detected first. The GO used in this work was prepared through a typical Hummers sonication method [35]. The thickness and lateral size of the GO sheet were 1.2–5 nm and 50 nm to 3 μm, respectively. Therefore, this ensured that the GO sheet was stretching rather than crimping into spherical particles [36]. XPS data (Figure S1d) showed the main elements of GO were C and O, and there was very little N. The functional groups of the GO sheet included the following percentages of the total carbon: carbonyl (C=O), 2.0%; hydroxyl (-OH), 46.5%; carboxyl (-C=(O)-OH), 2.5% (Figure 2a and Figure S1e). Those percentages imply that the graphene structure in GO sheet largely remained intact. The typical elements in BSA, HB, and Lyz other than C and O were N and S. Figure 2b,c shows the N 1s and S 2p XPS survey spectra of GO–BSA, GO–HB, and GO–Lyz NCF, further indicating the existence of BSA, HB, and Lyz. Moreover, the different atomic concentrations of each element in these films could also support the formation of these NCFs (Figure 2d). The polar binding sites of GO sheets usually existed on the edges and in the defects on the surface. Therefore, BSA/HB/Lyz was more likely to be bound on the surface of the GO sheet rather than the edge due to the huge difference between the size of any of these proteins and that of GO sheets.

**Figure 3:**
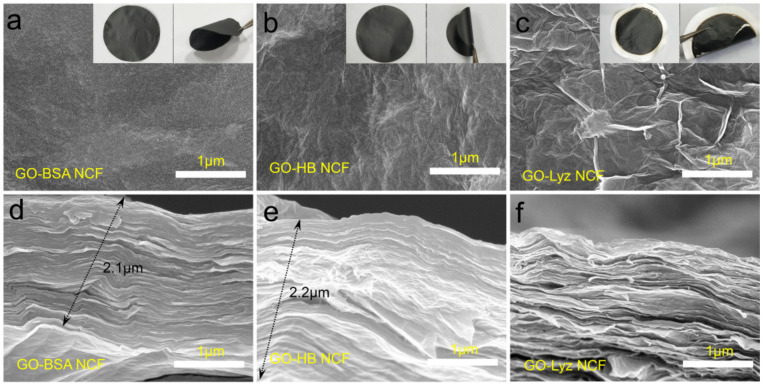
SEM images of surface (**a**–**c**); photographs with a uniform diameter of about 5 cm (insets of (**a**,**c**,**e**)). Internal cross-sections (**d**–**f**) of GO–BSA NCF, GO–HB NCF, and GO–Lyz NCF.

The insets in Figure S2a and Figure 3a–c show the megashapes of GO film, GO–BSA NCF, GO–HB NCF, and GO–Lyz NCF. GO–BSA and GO–HB NCFs had complete structures and exhibited bendability, whereas the GO film and GO–Lyz NCV were easily broken quickly. The reasons for this phenomenon were associated with the properties of these proteins and their binding situations with GO sheet. To further investigate the binding situations between the GO sheet and these proteins, SEM was employed to discover the surface microtopography. For the GO film, due to the polar sites on the surface and corner of the GO sheet, it was not easy to accomplish the *π*–*π* tight stacking in the GO sheets. That caused the GO film to have an uneven surface with great roughness (Figure S2a). On the contrary, GO sheet–BSA and GO sheet–HB compounds were achieved by adequate self-assembly, and their surfaces showed relatively good uniformity (Figure 3a,b). Thereinto, BSA and HB played the role of plasticizer to adjust the interfacial compatibility of GO. However, for the GO–Lyz NCF, its microscopic surface was the same as that of the GO film (Figure 3c), which meant the effect of this approach was negligible. These differences were also reflected in the GO sheet–protein compound solutions macroscopically. For both GO sheet–BSA and GO sheet–HB, the compound solutions, after a 16 h self-assembly process before suction filtration, were stable suspensions without precipitation or aggregation (Figure S3b,c). Most interestingly, both GO sheet–BSA and GO sheet–HB compound solutions were stable in polar aqueous solutions, which were more stable than the GO solution (Figure S3a), indicating that the external surfaces of GO sheet–BSA and GO sheet–HB compounds are also polar. Nevertheless, the external surface of GO sheet–Lyz compound was hydrophobic, and aggregation of the GO sheet–Lyz compound occurred through the spontaneous hydrophobic interactions, causing the apparent precipitation phenomenon of GO sheet–Lyz in the aqueous phase (Figure S3d). The stabilities of these compounds in aqueous solutions were closely related to the properties of the obtained NCFs.

SEM images of the internal cross-section were more valid evidence to confirm the above analysis. As shown in Figure 3d,e, both GO–BSA NCF and GO–HB NCF displayed a prominent layered hierarchical structure of natural nacre. Moreover, their compactness and smoothness in section micromorphology were better than those of GO and GO–Lyz NCFs when compared with the inter-layer gaps of the latter films (Figure 3f and Figure S2b). Although GO sheets were stable in the aqueous phase due to their polar sites, there were inevitably irregular gaps between GO sheets among the *π*–*π* tight stacking. By contrast, BSA and HB had better adhesion to GO sheets to facilitate the mutual attraction and fill the gaps for the dense structures. For Lyz, the structural change induced by the GO sheet was not beneficial for the tight and homogeneous binding of GO sheet–Lyz, and the instability of GO sheet–Lyz in an aqueous solution also caused inhomogeneity of GO–Lyz NCF. The excellent interfacial compatibility of GO sheet–protein compounds contributed to enhancing the mechanical properties of NCFs. Thereinto, the conformational changes of proteins induced by GO sheet were essential. Considering film-forming ability, GO–BSA NCF and GO–HB NCF are better candidates than GO–Lyz NCF for practical applications. Furthermore, using the SEM images of interior cross-sections, the thicknesses of GO–BSA and GO–HB NCF were determined to be around 2.1 and 2.2 μm, respectively.

The mechanical properties of GO–BSA and GO–HB NCF could also be reflected by the conformational changes of BSA and HB. To prove the secondary structure changes in BSA/HB induced by GO sheets, CD spectra of BSA and HB aqueous solutions with and without the addition of GO were obtained. The secondary structures (i.e., *α*-helix, *β*-pleated sheet, *β*-turn, and random coil) of proteins were confirmed by the positions of *α*-helixes (222 and 208 nm positive peaks, 192 nm negative peak), *β*-pleated sheets (217–218 nm positive peaks, 195–198 nm negative peak), *β*-turns (220–230 nm weak positive peaks, 180–190 nm strong positive peaks, 205 nm negative peak), and random coils (198 nm positive peaks, 220 nm negative peak) [37]. Figure 2e showed that there were apparent changes in the secondary structures of BSA and HB before and after binding of GO sheets, indicating that GO had the apparent effect on the structures of BSA and HB. After analyzing the data of contents of the secondary structures (Table 1), the changes in the secondary structures in BSA and HB showed the same pattern, which was a decrease in *α*-helixes and increases in *β*-pleated sheets (antiparallel and parallel), *β*-turns, and random coils. Before introducing GO sheets, *α*-helixes predominated in BSA and HB with the contents of 50.7 and 46.0%, respectively. After interactions with GO sheets, the *α*-helix contents of BSA and HB decreased to 14.6 and 16.4%, respectively. Our previous work has revealed the binding mechanism of the *α*-helix fragments of BSA with graphene by using molecular dynamics simulations [38]. The adsorption of an *α*-helix on the surface of graphene induces a transition from to the 3_10_-helix structure, which was reflected in the substantial increase in random coils from 24.1 to 42.2% for BSA in this work. This induction mode might also work for HB because the content of the random coils of HB increased from 26.5 to 41.4%. For the *β*-pleated sheets (antiparallel and parallel), the tiled state on GO sheets was more stable due to the interactions of more binding sites, which was in accordance with the molecular dynamics simulations of our previous work that showed graphene was advantageous to the stability of *β*-pleated sheets [39]. The increase in *β*-turns always accompanied the increase in *β*-pleated sheets. Therefore, the increases in contents of the *β*-pleated sheets (antiparallel and parallel) and random coils in BSA and HB were beneficial for the self-assembly of GO sheet with BSA and HB.

### 3.2. Stability of NCFs

The thermostability of films is very important to determine their applications at different temperatures. The thermostability of GO film, GO–BSA NCF, and GO–HB NCF was characterized by DSC. As shown in Figure 2f, heat release of the GO film proceeded the increase in temperature, indicating that the GO sheet was very sensitive to heat. After binding with BSA and HB, only transient heat release occurred in GO–BSA and GO–HB NCFs, and then the heat flows were maintained within a stable range, implying that GO–BSA and GO–HB NCF were thermostable. It is not difficult to see that BSA and HB also exhibited continuous heat release and absorption around 60–70 °C due to their conformational changes with the temperature change. The glass transition is the transition of amorphous material from a glassy state to a high elastic state. As shown in the inset of Figure 2f, the glass-transition temperatures of GO–BSA and GO–HB NCFs were confirmed to be 25.3 and 25.4 °C, respectively, which belong to the scope of room temperature. This indicates that the GO–BSA and GO–HB NCF could be kept in high elastic states at room temperature and remain stable. It was advantageous for GO–BSA and GO–HB NCFs to fully utilize their flexibility for stability. Before the self-assembly process of GO with BSA or HB, the conformational changes of BSA or HB were completed, so the film-forming process would not induce a conformational change in BSA or HB. That is to say, the film-forming process would only involve the self-assembly of “GO sheet-BSA/HB compounds.” Combined with the results of CD spectra, it could be concluded that the introduction of BSA or HB on the surface of a GO sheet was helpful to improving the thermostability, which is attributed to the increased contents of the *β*-pleated sheets (antiparallel and parallel) and random coils.

**Figure 4:**
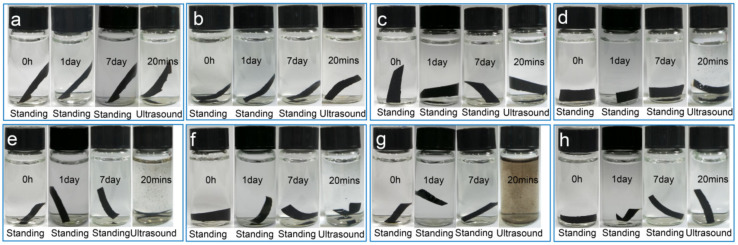
The stability of the GO–BSA NCF in aqueous (**a**), HCl (**b**), NaOH (**c**), and NaCl (**d**) solutions at room temperature. Stability of GO–HB NCF in aqueous (**e**), HCl (**f**), NaOH (**g**) and NaCl solution (**h**) with different treatments at room temperature.

The stability of films in various complex environments is also essential for their actual applications. It has been confirmed that the functional groups of the GO sheet were OH and –COOH. Even so, there were many hydrophobic areas on the surface of the GO sheet. The formation of the GO film was caused by the polar and non-polar interactions of GO sheets. The polar interactions included hydrogen bonding of –OH with –OH, –OH with –COOH, and –COOH with –COOH; non-polar interactions were *π*–*π* stacking. The GO film was barely stable in the aqueous phase and was dissolved partly after 7 days (Figure S4a). After ultrasonication, the mutual attraction of GO sheets could not conquer the destructive effect from outside that dissolved GO film easily. –COOH↔–COO^−^+H^+^ was a dynamic equilibrium process and water could break the hydrogen bonding to a certain degree. GO films were stable in acidic, alkaline, and saline environments for a standing time of 7 days (Figure S4b–d). However, they were unstable under ultrasonication in alkaline and saline environments because the films were dissolved easily, which we attribute to different dissolution mechanisms. The increase in –COO^−^ groups facilitated the electrostatic repulsion in an alkaline environment, and the saline ions destroyed the electrostatic attraction in a saline environment. Both cases were not beneficial for the stability the GO films. By contrast, GO films were still stable in an acidic environment, even under ultrasonication, because the increase in –COOH groups could tremendously enhance the mutual attraction of GO sheets. After determining the stability and instability mechanisms of GO films in the above environments, the assembly mechanisms of GO–BSA and GO–HB NCFs were also analyzed. The main functional groups of BSA and HB are –NH_3_, –COOH, hydrophobic chains (benzene ring and alkyl chain), and other polar groups (–OH, –C(=O)–NH–). The formation of GO–BSA and GO–HB NCFs meant self-assembly of GO sheet–BSA and GO sheet–HB compounds, respectively, which still involved multiple non-covalent interactions, as in the formation of GO films. The process was markedly different for BSA and HB, in types of interactions and binding strengths, owing to their uniqueness. Therefore, the stability of GO–BSA and GO–HB NCFs differed significantly in different environments. GO–BSA NCF was very stable in aqueous, acidic, alkaline, and saline environments with or without ultrasonic treatment, indicating that the favorable interactions for film-forming were far stronger than the adverse interactions (Figure 4a–d). There was no denying that the confirmation of interactions is very complex and needs precision instruments and testing [40,41]. GO–HB NCF was very stable in acidic and saline environments regardless of ultrasonic treatment (Figure 4f,h), but it can dissolve easily in aqueous and alkaline environments with ultrasonic treatment (Figure 4e,g). The stability of these films will determine their ranges of application, which is the case for all BMMs with a unique characteristics.

### 3.3. Mechanical Properties of NCFs

**Figure 5:**
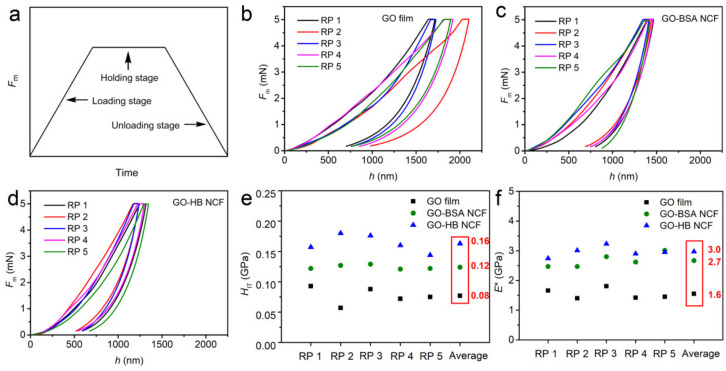
Typical scheme of nanoindentation load–displacement curve (**a**). Representative nanoindentation load–displacement curves for GO film (**b**), GO–BSA NCF (**c**), GO–HB NCF (**d**) with five RP. The corresponding *H*_IT_ (**e**) and E* (**f**) of the GO film, GO–BSA NCF, and GO–HB NCF.

The mechanical properties of GO film, GO–BSA NCF and GO–HB NCF were characterized by nanoindentation with five random positions (RP) under the maximum applied load (*F*_m_, mN) of 5 mN. Generally, there are three stages, namely, loading, holding, and unloading (Figure 5a), which are very apparent in *H*_IT_-*h* curves. There were significant differences in the tracks of five *H*_IT_-*h* curves of the GO films, indicating that the uniformity of GO films was relatively poor (Figure 5b), which is in accordance with the results of SEM in Figure S2a: the surfaces of GO films were uneven with large roughness. However, the tracks for GO–BSA and GO–HB NCFs (Figure 5c,d) were nearly identical, indicating that the uniformity of GO–BSA NCF and GO–HB NCF is much better than that of GO film. In addition, the corresponding indentation hardness (*H*_IT_, GPa) and equivalent elastic modulus (E*, GPa) of each *H*_IT_-*h* curve can be calculated. Figure 5e,f shows the *H*_IT_ and E* of each RP of a GO film, GO–BSA NCF, and GO–HB NCF, and there are no abrupt values, indicating these films had good structural homogeneity and no flaws. Numerous studies have strived to improve the mechanical properties of GO-based films because their limited mechanical properties have hindered practical applications [42,43,44]. Here, the average *H*_IT_ and E* of GO–BSA NCF were 0.12 and 2.7 GPa, respectively; the average *H*_IT_ and E* of GO–HB NCF were 0.16 and 3.0 GPa, respectively. Compared with average the *H*_IT_ (0.08 GPa) and E* (1.6 GPa) of GO film, the mechanical properties of GO–BSA and GO–HB NCF were very much improved. Under *F*_m_ of 5 mN, the depths of indentation of GO–BSA and GO–HB NCF were around 1300 and 1200 nm, which were lower than that of GO film (1600–2000 nm), indicating that the existence of BSA and HB in interlayers of films could store the stress. BSA and HB served as filling agents and plasticizers to complement the stretchability of GO. In the work of Li et al., the functional groups on GO sheets had a significant influence on the mechanical properties of a GO–silk-based nanocomposite, and the oxygen-containing groups of GO could form hydrogen bonding with silk fibroins at the interface to improve the adhesive force [45]. A similar principle applies for BSA and HB, because BSA or HB could bind to the surface of a GO sheet strongly through hydrogen bonding, in addition the spontaneous hydrophobic interactions, which would shield the sheet from water molecules surrounding GO–BSA NFC or GO–HB NCF, thereby stabilizing their structures [45]. Therefore, the resistance to instantaneous and continuous external forces in GO–BSA and GO–HB NCF is significantly improved over that of GO films, meaning that GO–BSA and GO–HB NCFs exhibit enhanced applicability. In the previous work of Shao and Fan et al., bacterial cellulose and chitosan were fabricated with GO to improve the mechanical properties of GO–bacterial cellulose and GO–chitosan NCF, respectively, and the assembly process only involved hydrogen bonding and electrostatic interactions [46,47]. Compared with Shao’s work with an E* of 0.5 GPa, GO–BSA NCF and GO–HB NCF had significant advantages in terms of mechanical properties [46]. In addition, although the *H*_IT_ (0.40 GPa) and E* (6.5 GPa) of GO–chitosan NCF in Fan’s work were much higher than those of GO–BSA and GO–HB NCF, GO–chitosan NCF was extremely unstable in an acidic environment, which will handicap its applicability [47]. Therefore, combining GO and well matched BMMs is an effective and fantastic strategy to construct nacre-like NCFs with good stability and enhanced mechanical properties.

## 4. Conclusions

In summary, GO–BMM-based NCFs with nacre-mimetic structures were fabricated with GO and proteins through a green and straightforward non-covalent self-assembly process. The sequences and conformational features of BSA, HB, and Lyz determined their film-forming ability with GO sheets, implying that GO–BSA and GO–HB, which are stable compounds in an aqueous solution, are outstanding candidates for fabricating stable NCFs. The GO sheet induced increases in the presence of *β*-pleated sheets, *β*-turns, and random coils in BSA and HB, along with a decrease in the presence *α*-helixes, which was more beneficial for the NCFs with dense and uniform microstructures. Compared with the GO film, GO–BSA and GO–HB NCF exhibited good thermostability below 100 °C, and remained remarkably stable in acidic and saline environments. GO–BSA NCF could be kept stable in an alkaline environment, which endows it with broader application potential. Moreover, GO–BSA and GO–HB NCF exhibited significant advantages through appreciable HIT enhancements of 50.0% and 100%; and enhancements in E* by 68.6% and 87.5%, respectively. The binding of BMMs into interlayers of 2D nanomaterials could synergistically provide enhancements while maintaining the films’ respective characteristics, making them promising for flexible devices.

## Figures and Tables

**Table 1:** The contents of the secondary structure elements of BSA and HB with and without GO.

Secondary Structure		BSA (%)	BSA in the Presence of GO Sheet (%)	HB (%)	HB in the Presence of GO Sheet (%)
*α*-Helix		50.7	14.6	46.0	16.4
*β*-Pleated sheet	Antiparallel	5.6	11.6	5.8	11.1
	Parallel	5.2	14.3	6.5	13.7
*β*-Turn		14.4	17.3	15.2	17.4
Random coil		24.1	42.2	26.5	41.4

## Data Availability

The data presented in this study are available on request from the corresponding author.

## Supplementary Materials

The following supporting information can be downloaded at: https://www.mdpi.com/article/10.3390/nano12071181/s1. Figure S1: The crystal structures of BSA (a, PDB code 4F5S), HB (b, PDB code 1FN3), and Lyz (c, PDB code 253L) with the style of newcartoon; wide scan of XPS survey spectra of films (d); atomic concentration of C in GO film (e). Figure S2: SEM images of surfaces (a); photographs with uniform diameter of about 5 cm (insets of a). Internal cross section (b) of GO film. Figure S3: Optical microscope photographs of the GO solution (a), GO–BSA compound solution (b), GO–HB compound solution (c) and GO–Lyz compound solution (d); Figure S4: The stability of GO film in aqueous (a), HCl (b), NaOH (c), and NaCl (d) solutions at room temperature.
