# Supplementary material for: Enhanced Stability and Mechanical Properties of a Graphene–Protein Nanocomposite Film by a Facile Non-Covalent Self-Assembly Approach
Source: Nanomaterials (Basel). 2022 Apr 1;12(7):1181. doi: 10.3390/nano12071181 (PMC9000757; doi:10.3390/nano12071181)
Supplement: Supplementary file 1 [file nanomaterials-12-01181-s001.zip › nanomaterials-1645637-supplementary.pdf]

nanomaterials

MDPI

Supplementary Materials

Enhanced Stability and Mechanical Properties of a Graphene–Protein Nanocomposite Film by a Facile Non-Covalent Self-Assembly Approach

Chunbao Du [1], Ting Du [1], Joey Tianyi Zhou [3], Yanan Zhu [1], Xingang Jia [1] and Yuan Cheng [2,4,*]

[1] College of Chemistry and Chemical Engineering, Xi'an Shiyou University, Xi'an 710065, China; duchunbao218@126.com (C.D.); duting102@126.com (T.D.); zhuyanan@xsyu.edu.cn (Y.Z.); jiaxingang76@xsyu.edu.cn (X.J.)
[2] Monash Suzhou Research Institute, Monash University, Suzhou Industrial Park, Suzhou 215000, China
[3] Institute of High Performance Computing, A*STAR, Singapore 138632, Singapore; joey_zhou@ihpc.a-star.edu.sg
[4] Department of Materials Science and Engineering, Monash University, Clayton, VIC 3800, Australia
* Correspondence: yuan.cheng@monash.edu

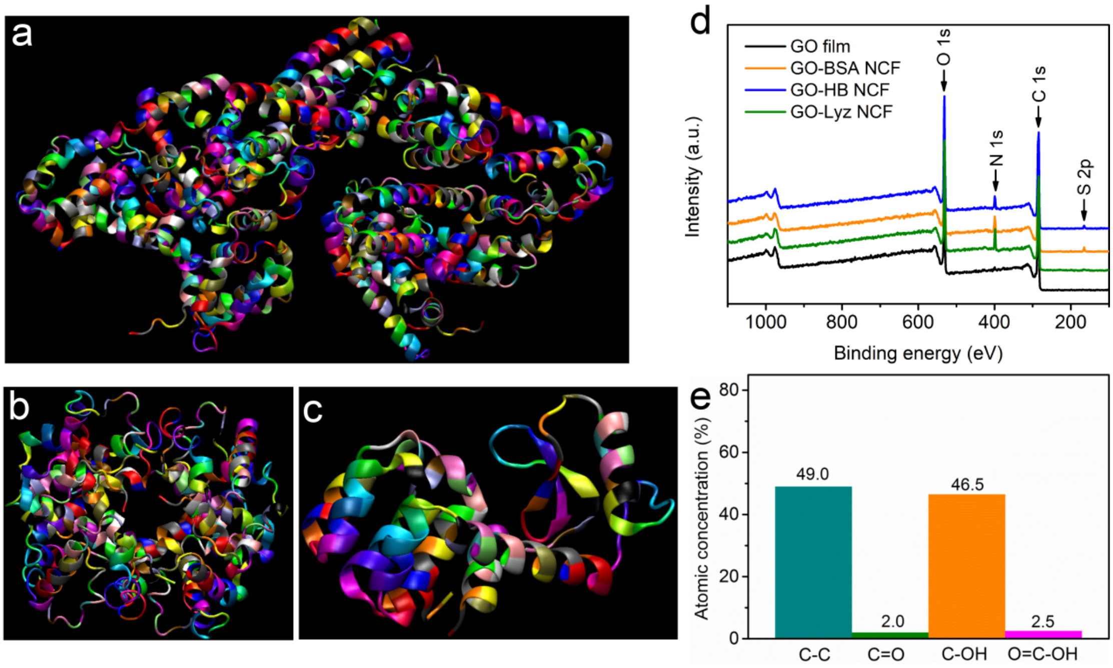

Figure S1. The crystal structures of BSA (a, PDB code 4F5S), HB (b, PDB code 1FN3) and Lyz (c, PDB code 253L) with the style of newcartoon; wide scan of XPS survey spectra of films (d); atomic concentration of C in GO film (e).

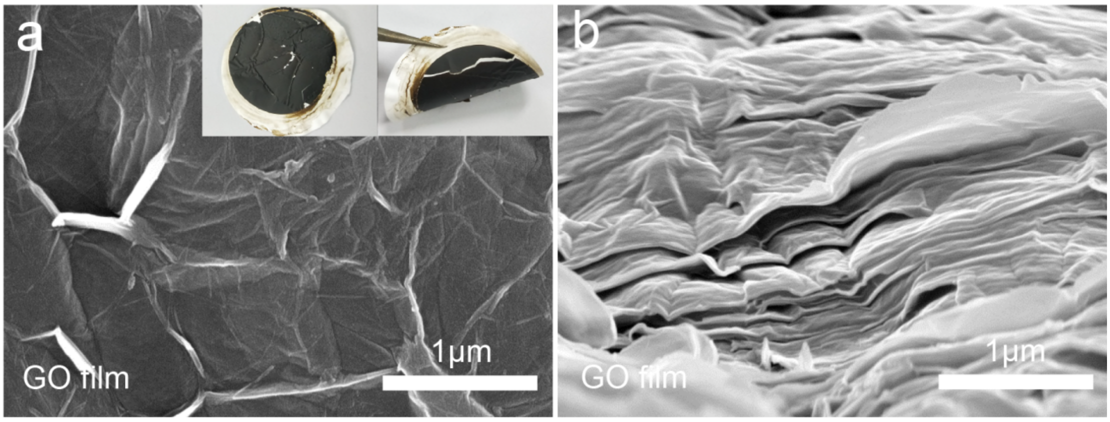

Figure S2. SEM images of surface (a), photographs with uniform diameter of about 5 cm (insets of a) and internal cross section (b) of GO film.

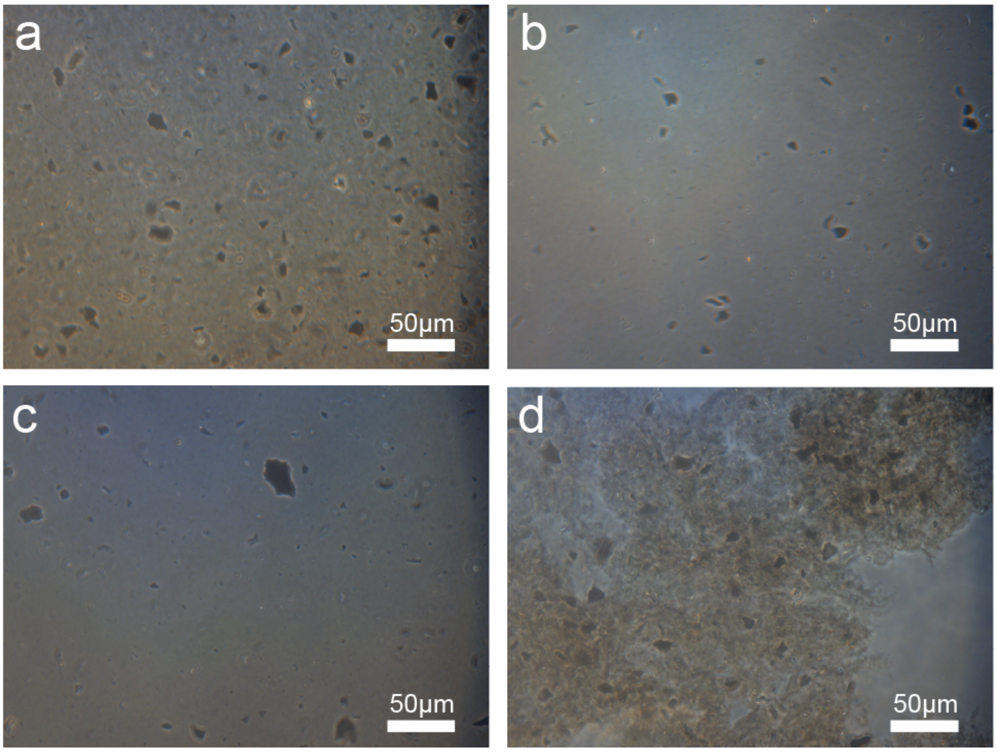

Figure S3. Optical microscope photographs of GO solution (a), GO-BSA compound solution (b), GO-HB compound solution (c) and GO-Lyz compound solution (d).

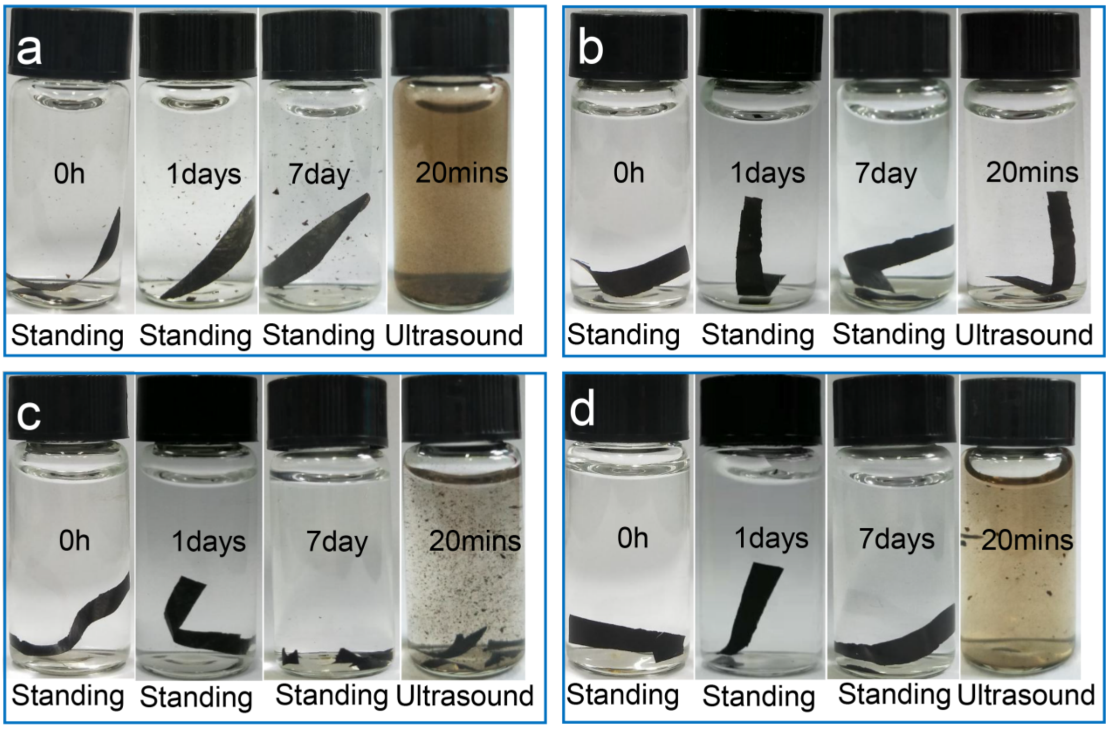

Figure S4. The stability of GO film in aqueous (a), HCl (b), NaOH (c) and NaCl solution (d) with different treatment at room temperature.
